# Determination of citrate content in specialty paper based on headspace gas chromatography

**DOI:** 10.3389/fchem.2025.1643516

**Published:** 2025-07-04

**Authors:** Qiyu He, Baoshan Yue, Xinruitong Liu, Hongyue Zhu, Zhenhua Yu, Ying Zhang, Cong Gao, Wei Chen, Yingchun Luo, Jiao Xie, Yi Dai

**Affiliations:** ^1^ School of Chemical Engineering, Guizhou Minzu University, Guiyang, China; ^2^ Technology Center, China Tobacco Yunnan Industrial Co. Ltd., Kunming, China

**Keywords:** citrate, specialty paper, headspace, GC, combustion promoters

## Abstract

A method for determining citrate content in specialty paper using headspace gas chromatography (HS-GC) is proposed. This method is based on the reaction between sodium citrate and potassium permanganate under acidic conditions, which generates CO_2_. The CO_2_ is detected by a thermal conductivity detector and the sodium citrate content is calculated using a standard curve. Optimization of the method was conducted by investigating various parameters, including gas chromatography conditions, equilibrium time, equilibrium temperature, and injection volume. The method’s accuracy and precision were assessed through method validation. The results demonstrated that the relative standard deviation (RSD) was ≤3.00%, and the recovery rate ranged from 91% to 102%, indicating good reliability and accuracy. This method is simple, rapid, and precise, making it an effective approach for the determination of citrate content in specialty paper.

## 1 Introduction

Specialty paper refers to paper enhanced with various additives to impart specific functions (Li et al., 2021). It is widely used in fields such as medicine (Liu et al., 2020), batteries (Stamatelos et al., 2023), tobacco (Gao et al., 2015), food packaging (Shan, 2021), and automotive electronics (Qijie et al., 2013). Cigarette paper, a type of specialty paper, benefits from the addition of combustion promoters (Jo et al., 2024). These additives not only improve the ash condensation ability and enhance ash retention (Zheng et al., 2021), but also regulate the combustion rate to improve cigarette quality (Li et al., 2010). Most importantly, combustion promoters can reduce the distinct odor and spiciness of cigarettes while lowering CO content (Yamamoto et al., 1990). However, the concentration of combustion promoters must be carefully controlled (Zhao et al., 2013). Excessive amounts may cause a mismatch between the combustion rates of the cigarette paper and tobacco, negatively affecting smoking quality. Since citrate is the most commonly used combustion promoter in cigarette paper (Resnik et al., 1977), rapid detection of its content is important.

Common methods for detecting citrate include redox titration (Meija et al., 2017), Ion chromatography (IC) (Jackson, 2000), inductively coupled plasma mass spectrometry (ICP-MS) (Wilschefski and Baxter, 2019; Veillon and Margoshes, 1968), and atomic absorption spectrometry (AAS) (Welz and Sperling, 2008). Redox titration involves using potassium permanganate for a color reaction to determine citrate content based on the consumption of the permanganate (Dybko et al., 1995). This method is complex and subject to significant operator variability in determining the endpoint. IC is an efficient chromatographic technique (Rybakova, 2023) that separates substances based on their mobility in ion exchange columns, followed by automatic detection (Jackson, 2000). It is widely used for anion and cation analysis. ICP-MS atomizes the sample, dissociates it with argon plasma, and detects it with a mass spectrometer (Wilschefski and Baxter, 2019). While this method reduces subjective error, it requires extensive sample pretreatment, which is time-consuming and may introduce residual effects on the results. The principle of AAS is based on the absorption of characteristic radiation emitted by the source, specific to the element being analyzed, by gaseous ground-state atoms of that element in the vapor phase. The concentration of the element in the sample is determined from the extent of this radiation attenuation (Welz and Sperling, 2008). While AAS is one of the most commonly used analytical techniques (Ferreira et al., 2018), it is generally applicable to the detection of metallic elements. Consequently, the citrate concentration can only be inferred indirectly from the measured Na^+^ concentration, a process which involves a more complex procedure.

Headspace gas chromatography (HS-GC) is an effective method for determining volatile components in complex matrix samples (Dai et al., 2021; Dai et al., 2019; Zhang et al., 2015) When the liquid or solid analyte reaches equilibrium in a sealed headspace vial, the top gas is extracted for analysis by gas chromatography (Lim and Shin, 2016). This approach minimizes interference from other complex components that may affect results when sampling directly from liquid or solid samples (Zhu and Chai, 2005). HS-GC is simple, rapid, and accurate, making it widely used in food processing, medical, chemical, and other industries (Chai and Zhu, 2000).

This paper presents a headspace gas chromatography method for measuring CO_2_ in headspace vials, using cigarette paper as an example. The method is based on the reaction between sodium citrate and potassium permanganate, which generates CO_2_ under acidic conditions. The content of sodium citrate in cigarette paper is then determined by measuring the CO_2_. The key focus of this study is the optimization of various parameters, including gas chromatography conditions, equilibrium time, equilibrium temperature, and injection volume. The accuracy and precision of the method were also evaluated.

## 2 Materials and methods

### 2.1 Materials

All chemicals used in the experiment—sodium citrate, sulfuric acid, and potassium permanganate—were of analytical grade. Deionized water was prepared in the laboratory.A 0.1 mol/L potassium permanganate (KMnO_4_) solution was prepared by accurately weighing 1.58 g of solid KMnO_4_ powder using an electronic balance. The powder was dissolved in a minimal volume of distilled water and transferred via glass rod into a 100 mL volumetric flask. The beaker was subsequently rinsed multiple times with small portions of distilled water, with each rinsate transferred to the flask using the glass rod. The solution was then diluted to the mark with distilled water and thoroughly mixed. A 20% dilute sulfuric acid solution was prepared by measuring 80 mL of distilled water into a 250 mL beaker. This beaker was placed in a 1,000 mL beaker containing an appropriate amount of water (acting as a cooling bath). Concentrated sulfuric acid (w/% = 95%–98%, 20 mL) was added cautiously in small aliquots to the distilled water with continuous stirring using a glass rod. The sodium citrate standard stock solution (1.94 × 10^−3^ mol/L) was prepared by accurately weighing 0.05 g of sodium citrate on an electronic balance. The solid was dissolved in distilled water and quantitatively transferred to a 100 mL volumetric flask, followed by dilution to the mark.

### 2.2 Apparatus and operations

The headspace gas chromatography system consists of an automatic headspace sampler (DANI HS 86.50 PLUS, Italy) and a gas chromatography system with a thermal conductivity detector (TCD) (Agilent GC 8860, United States) Headspace conditions included 20 min of equilibration at 80°C with vigorous shaking. The quantitative ring temperature is 90°C, and the transmission line temperature is 100°C. The pressurized pressure is 0.73 bar, the carrier gas pressure is 0.68 bar, the carrier gas balance time in the headspace sample bottle is 0.2 min, the pipeline inflation time is 0.2 min, and the pipeline balance time is 0.2 min. The quantitative ring volume is 3.0 mL, and the volume of each headspace sample bottle is 20.0 mL. The gas chromatography operating conditions are as follows: carrier gas is nitrogen with a flow rate of 2 mL/min, column flow rate is 15.06 mL/min, injection port temperature is 250°C, column box temperature is 95°C, and the DB-5 capillary column specifications are 30 m × 0.32 mm × 0.25 μm. The detection time is 3 min, with a split ratio of 0.1:1.

### 2.3 Sample preparation

To prepare the cigarette paper leaching solution, Cigarette paper (2 g) was accurately weighed using an electronic analytical balance, cut into 2 cm × 2 cm fragments, and transferred into a conical flask. Then, 100 mL of distilled water was added, and the flask was placed on an oscillator. The mixture was shaken at 130 r/min for 90 min. A small amount of 20% dilute sulfuric acid was added in increments until no bubbles were produced. Following this, 50 mL of 20% dilute sulfuric acid was added, and the supernatant was filtered into a reagent bottle for later use.

### 2.4 Experimental methods

**FIGURE 1:**
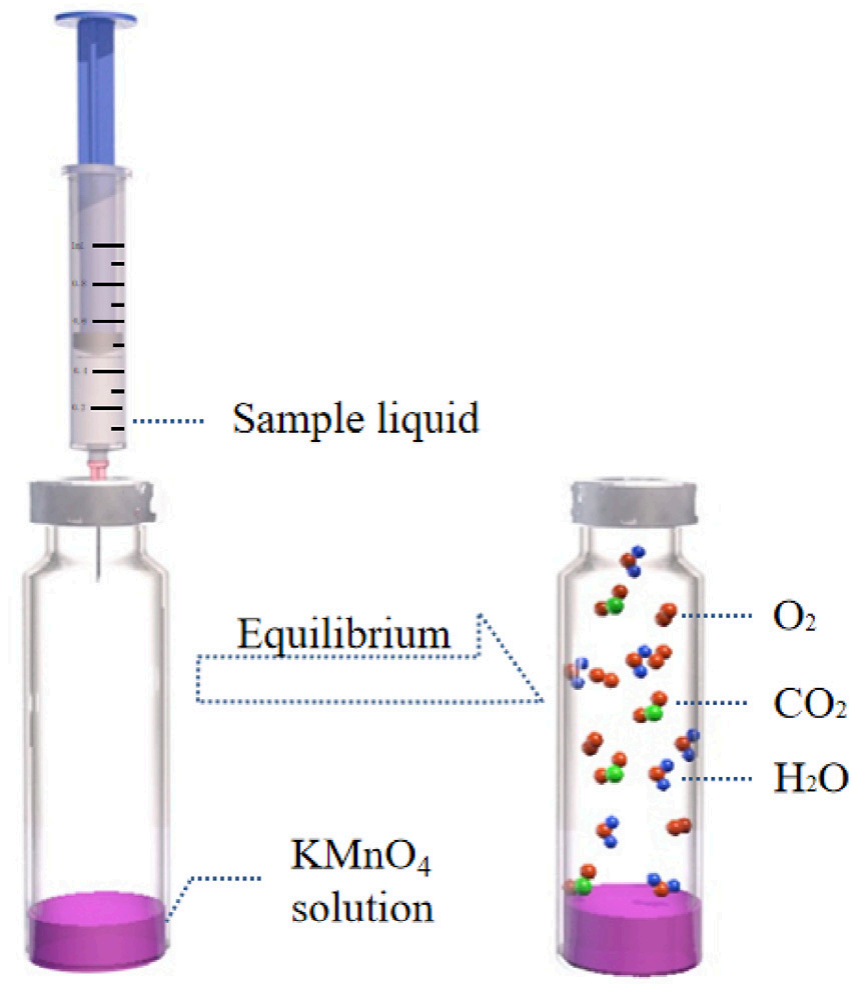
Reactive principle sketch.

1 mL of potassium permanganate solution was transferred into a headspace bottle using a pipette, and the bottle was sealed with an aluminum cap fitted with a polytetrafluoroethylene silicone rubber gasket. Then, 0.5 mL of cigarette paper leaching solution was injected into the headspace bottle using a syringe. The mixture was shaken thoroughly to ensure the leaching solution reacted with the potassium permanganate under acidic conditions. The headspace bottle was then placed into the headspace sampler for HS-GC detection. The schematic as shown in Figure 1.

## 3 Results and discussion

### 3.1 Experimental principles

**FIGURE 2:**
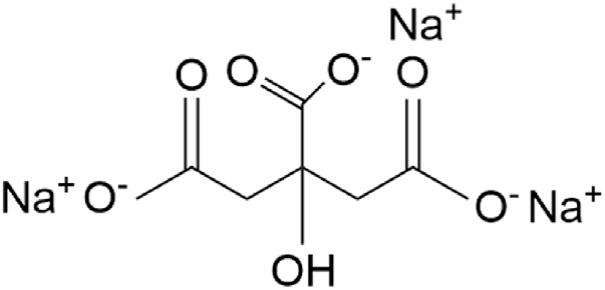
The chemical structural formula of sodium citrate.

This method determines the content of sodium citrate by measuring the CO_2_ generated from the reaction of sodium citrate (chemical structure formula as shown in Figure 2) and potassium permanganate under acidic conditions. The specific reaction is shown in Equation 1:

$${\text{Mn}}_{4}^{-}+{\text{COO}}^{-}+H^{+}\to {\text{Mn}}^{2+}+{\text{CO}}_{2}\left(g\right)+H_{2}O$$ (1)

During the headspace gas chromatography analysis, a portion of pressurized N_2_ gas is injected into a closed headspace bottle, and the gas phase is released into the atmosphere through the sample ring. The connection of the sample ring is then switched, allowing the headspace sample to be injected into the GC system for measurement (Dai et al., 2017).

**FIGURE 3:**
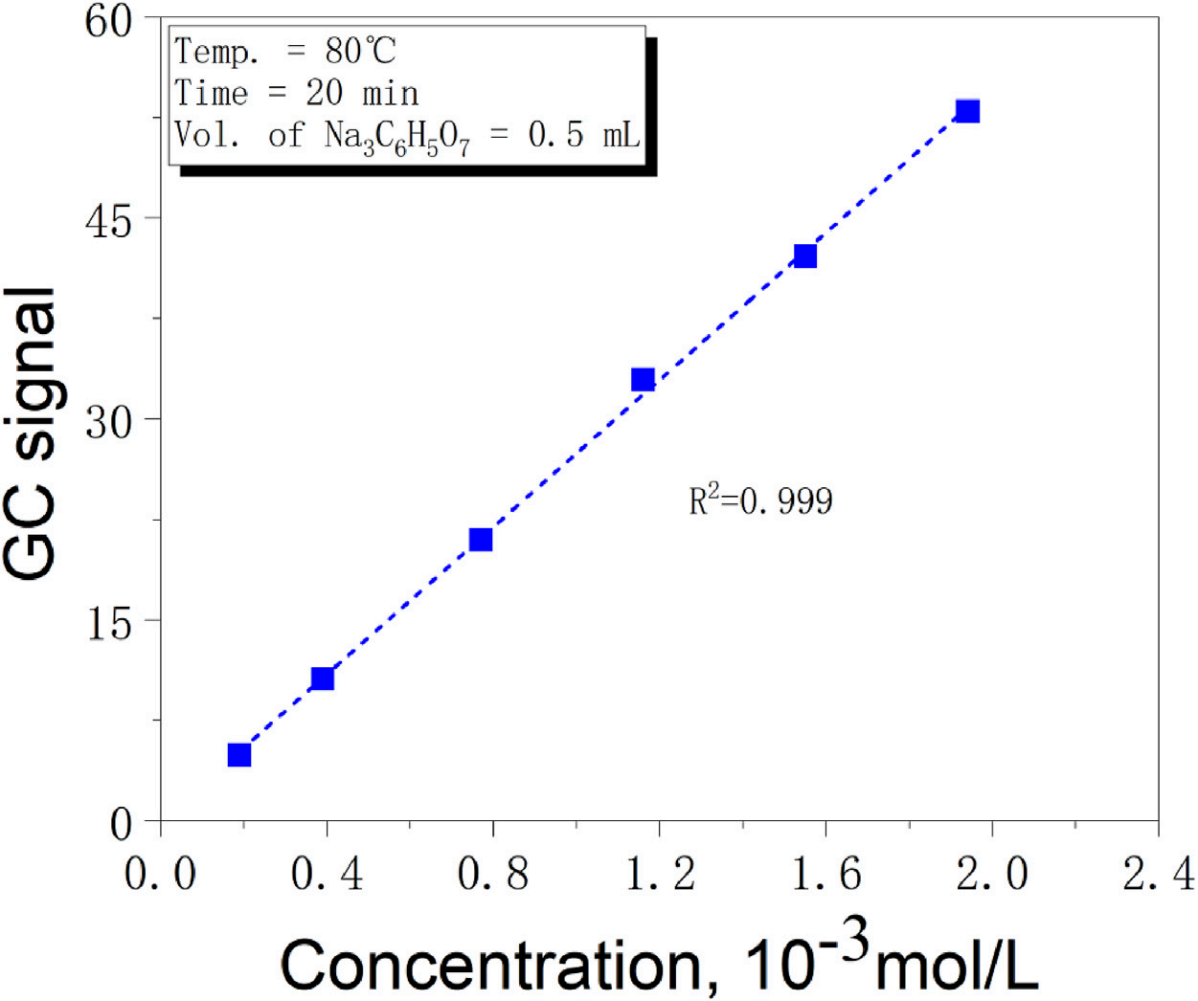
The relationship between the content of sodium citrate and GC signal value.

Pipettes were used to transfer 1, 2, 4, 6, and 8 mL of the standard stock solution into 10 mL volumetric flasks, which were then diluted to the mark with distilled water to obtain standard solutions at concentrations of 0.19 × 10^−3^, 0.39 × 10^−3^, 0.77 × 10^−3^, 1.16 × 10^−3^, 1.55 × 10^−3^, and 1.94 × 10^−3^ mol/L. A standard curve was constructed by measuring the standard solutions, as shown in Figure 3. The standard curve reveals a strong linear relationship between the GC signal value of CO_2_ detected by HS-GC and the sodium citrate concentration. Based on this, the sodium citrate content in cigarette paper can be determined by measuring the CO_2_ signal value in the headspace bottle using the HS-GC method.

### 3.2 Optimization of gas chromatography measurement conditions

**FIGURE 4:**
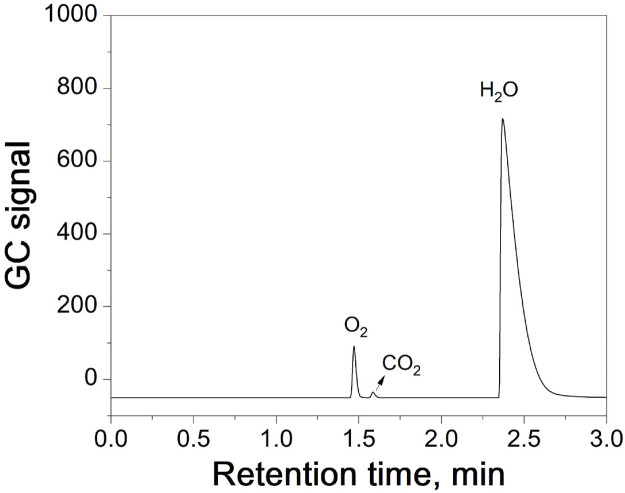
Chromatogram of a sample from HS-GC testing.

Figure 4 shows the chromatogram of the sample detected using HS-GC. When N_2_ is used as the carrier gas, the gas chromatograph detects O_2_, CO_2_, and H_2_O. As seen in the figure, their peaks are well separated under the current gas chromatography conditions (RSD < 4.00%).

### 3.3 Optimization of headspace equilibrium conditions

**FIGURE 5:**
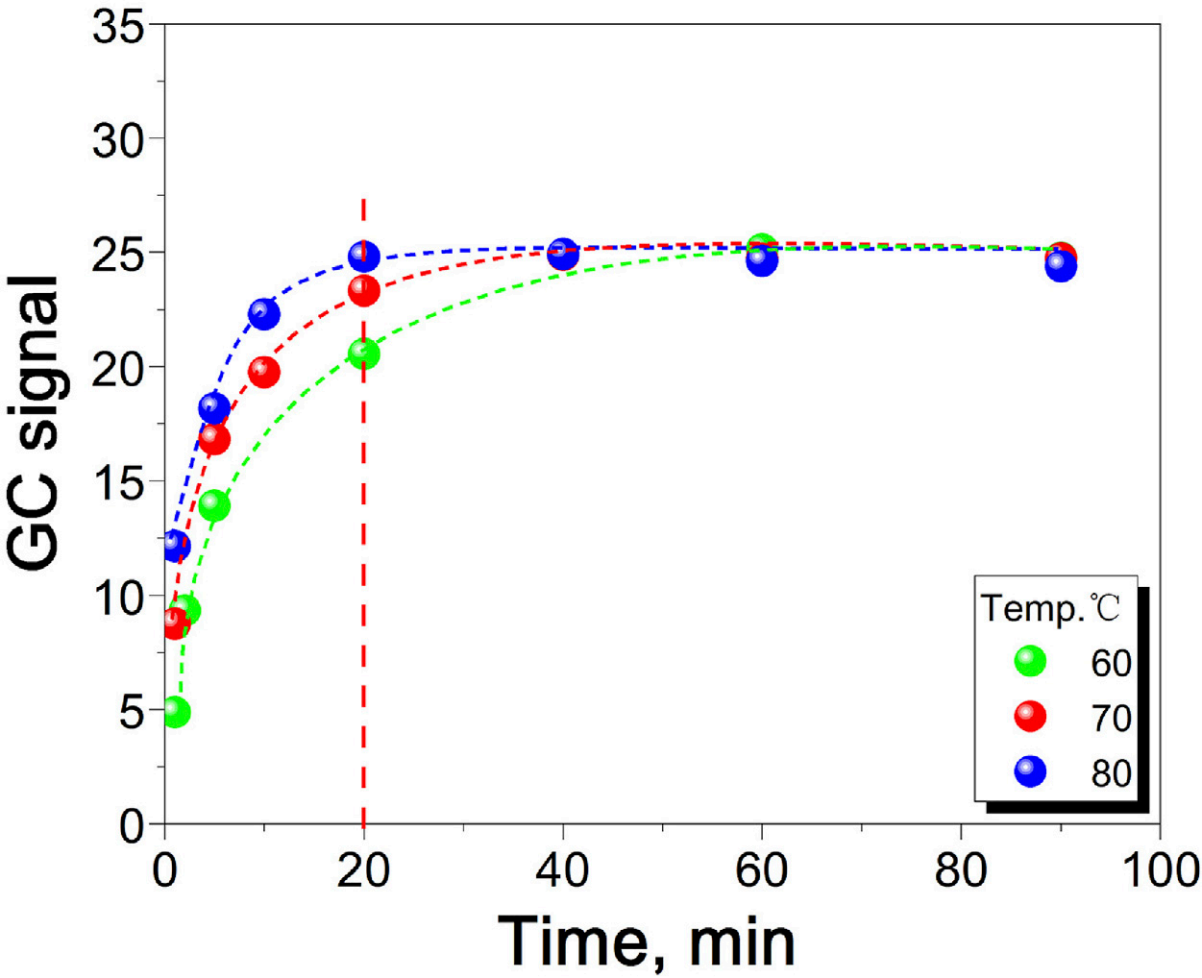
The relationship between equilibrium time, equilibrium temperature and GC signal value.

A higher equilibrium temperature leads to a shorter equilibrium time, faster reaction rate, and increased experimental efficiency (Chen et al., 2020; Zhu et al., 2024). However, in the experiment, shortening the test cycle is not the only consideration, and it is also necessary to ensure the complete reaction in the headspace bottle and the safety of the headspace bottle reaction. Therefore, it is particularly important to find the appropriate equilibrium time and equilibrium temperature (Zhu et al., 2025). Figure 5 shows the effect of equilibrium time and equilibrium temperature on GC signal value. It can be seen from the figure that when the equilibrium temperature is 60°C and 70°C, the time required for the reaction to reach equilibrium is longer, which is 40 and 60 min, respectively. When the equilibrium temperature is 80°C, the reaction reaches equilibrium at 20 min. At the same time, in order to prevent the water peak from being too high, resulting in leakage of the headspace bottle, the temperature should not be too high. Therefore, in this experiment, 80°C and 20 min were selected as the equilibrium temperature and equilibrium time.

### 3.4 Optimization of sample injection volume

**FIGURE 6:**
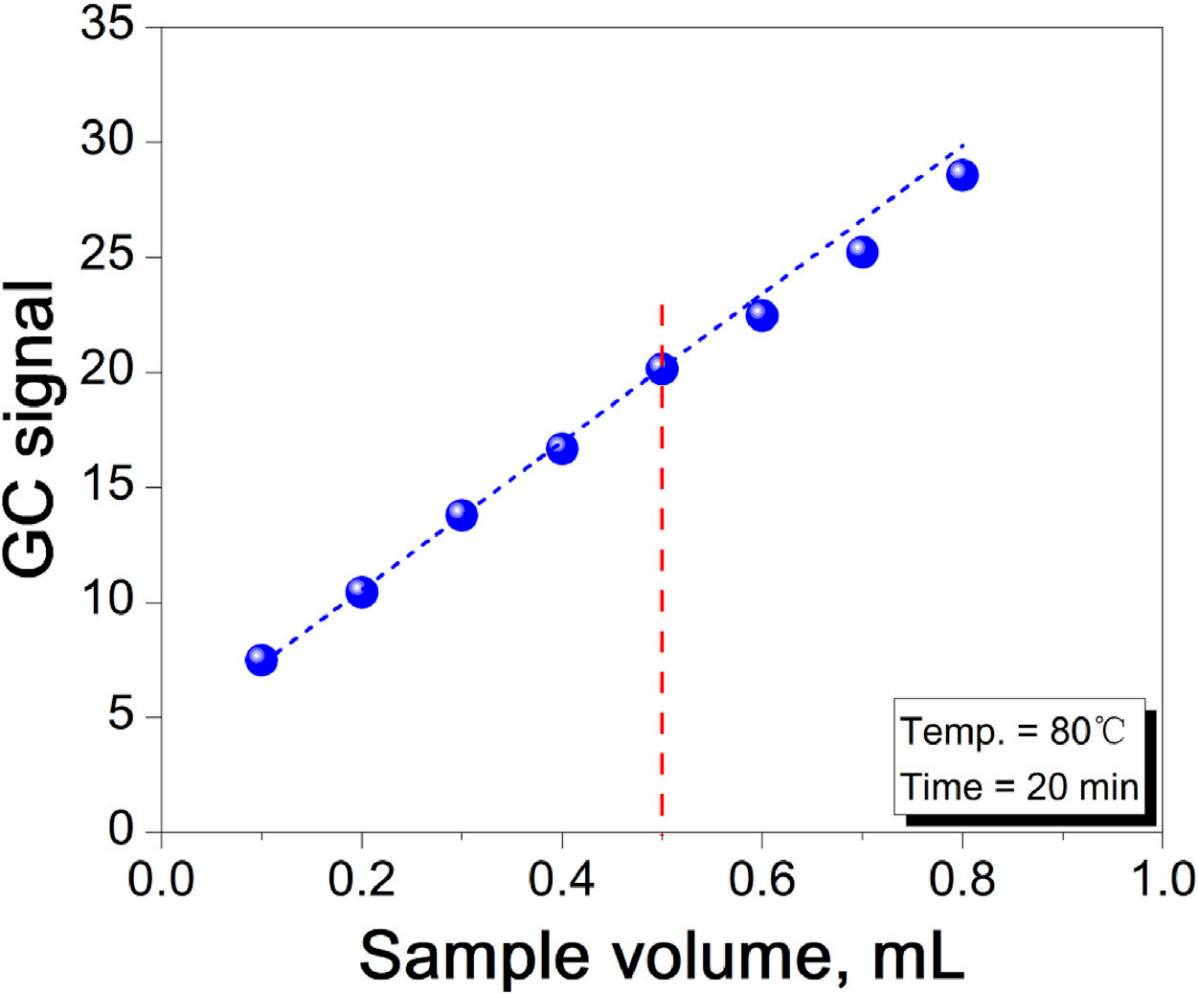
The effect of injection volume on GC signal value.

Increasing the sample injection volume enhances the sensitivity of headspace analysis (Zhang et al., 2014), but excessive injection volume can lead to several issues. First, a larger injection volume increases the time required for the reaction to reach equilibrium, extending the experimental cycle and conflicting with the principles of green chemistry. Second, an overly large injection volume can cause excessive pressure in the headspace bottle during the reaction, risking leakage or even cracking. Therefore, it is crucial to select an appropriate injection volume. Figure 6 illustrates the effect of injection volume on the GC signal value in headspace gas chromatography. The linear relationship remains strong up to a 0.5 mL injection volume, beyond which it begins to deteriorate. Considering the need to avoid excessive injection volume, 0.5 mL was chosen for this experiment.

### 3.5 Method evaluate

#### 3.5.1 Method calibration

Take 1 mL of 0.1 mol/L potassium permanganate solution and add it to a headspace bottle. Then, add 1 mL of 20% dilute sulfuric acid into the same bottle. After sealing the bottle with the cap, inject 0.5 mL of the standard solution with a gradient concentration using a syringe. The headspace bottle is then shaken and placed in the HS-GC for measurement to obtain the standard curve. The results show a strong linear relationship between the GC signal value of CO_2_ and the concentration of sodium citrate. The linear relationship can be expressed as Equation 2:

$$A=27.382\left(\pm 0.416\right)+0.0215\left(\pm 0.489\right)\;\left(n=6,R^{2}=0.999\right)$$ (2)

The limit of quantitation (LOQ) of this method for the detection of sodium citrate content can be calculated by Equation 3:

$$LOQ=\frac{a+10\left|\Delta a\right|}{s}$$ (3)

In the formula, a, s and Δa represent the intercept, slope, and standard deviation of the intercept from Equation 2, respectively. The quantitative limit of this method for detecting sodium citrate in cigarette paper can be calculated using Equation 3, yielding a value of 0.18 × 10^−3^ mol/L.

#### 3.5.2 Precision and validation evaluation

Precision is a key parameter for evaluating the uncertainty of experimental results and is essential for ensuring accuracy. In this experiment, three different cigarette paper samples underwent five sets of repeated tests under the optimized conditions, as shown in Table 1. The relative standard deviation (RSD%) for all three sample groups was ≤3.00%, indicating that the method demonstrates good precision.

**TABLE 1:** Repeatability of the method.

Replica no.	GC signal		
	Sample 1	Sample 2	Sample 3
1	34.87	30.23	24.81
2	34.92	29.48	24.85
3	33.72	30.67	24.81
4	34.66	28.41	25.63
5	34.94	29.17	25.44
Average	34.62	29.59	25.11
RSD (%)	1.56	3.00	1.58

To verify the accuracy of the method, a recovery experiment was conducted. Standard solutions of 0.39, 0.77, 1.16, 1.55, and 1.94 × 10^−3^ mol/L were added to five samples. The concentration of sodium citrate was calculated based on the GC signal value measured under optimized conditions, and the concentration of sodium citrate in the sample without the standard was subtracted. The recovery rate was determined by the ratio of the calculated concentration a to the initial concentration b. The results in Table 2 show that the recovery rate of sodium citrate ranges from 91% to 102%, indicating that the method has good accuracy.

**TABLE 2:** Method validation.

Sample ID	The content of sodium citrate (10^−3 ^mol/L)			RSD (%)
	Added (a)	Measured (b)	Recovery (%)	
1	0.39	0.43	91	3.06
2	0.77	0.81	96	2.98
3	1.16	1.13	103	2.37
4	1.55	1.56	99	3.61
5	1.94	1.90	102	3.07

To assess the reliability of the reaction system, the prepared potassium permanganate (KMnO_4_) solution was placed in the bottom compartment of a headspace vial. Using a syringe, 0.5 mL of sodium citrate solution (containing excess acid) was injected into the vial, followed by vortex mixing. The vial was then equilibrated at 80°C for durations of 1, 5, 10, 20, and 60 min prior to HS-GC analysis. Triplicate experiments were performed for each time point. As shown in Table 3, all relative standard deviations (RSD) were below 5%, demonstrating that the reaction solution exhibits excellent stability throughout the analytical cycle.

**TABLE 3:** Stability test of reaction solution.

Equilibrium time (min)	GC signal			RSD (%)
	Sample 1	Sample 2	Sample 3	
1	11.77	11.99	12.72	4.09
5	17.54	18.17	18.11	1.94
10	19.89	21.45	22.29	2.80
20	24.81	24.85	25.63	1.84
60	24.76	25.41	24.93	1.35

## 4 Conclusion

This study presents a rapid quantitative method for determining citrate content in specialty papers based on headspace gas chromatography (HS-GC). By innovatively leveraging the quantitative generation of CO_2_ from citrate under acidic potassium permanganate (KMnO_4_) conditions, this work achieves for the first time highly selective indirect detection of the target analyte within complex paper-based matrices. Optimization studies identified 80°C and 20 min as the optimal equilibrium temperature and time, respectively, with a 0.5 mL headspace injection volume proving most suitable. The method demonstrates superior precision (RSD ≤3.00%) and accuracy (standard addition recovery rate: 91%–102%), significantly outperforming conventional techniques. Crucially, by directly detecting gaseous CO_2_, this approach effectively circumvents interference from pulp matrices. Combined with its solvent-free green pretreatment workflow, it offers the specialty paper industry—particularly for combustion regulator control in cigarette paper—an efficient, economical, and reliable quality control solution. Furthermore, its operational simplicity, rapid analysis, high precision, and accuracy not only provide key technical support for optimizing paper functional performance (e.g., combustion modulation, CO reduction) but also establish a novel approach for detecting anionic additives in paper-based materials (Li et al., 2023).

## Data Availability

The original contributions presented in the study are included in the article/supplementary material, further inquiries can be directed to the corresponding authors.
